# Metabolism of Citrate and Other Carboxylic Acids in Erythrocytes As a Function of Oxygen Saturation and Refrigerated Storage

**DOI:** 10.3389/fmed.2017.00175

**Published:** 2017-10-17

**Authors:** Travis Nemkov, Kaiqi Sun, Julie A. Reisz, Tatsuro Yoshida, Andrew Dunham, Edward Y. Wen, Alexander Q. Wen, Rob C. Roach, Kirk C. Hansen, Yang Xia, Angelo D’Alessandro

**Affiliations:** ^1^Department of Biochemistry and Molecular Genetics, University of Colorado Denver – Anschutz Medical Campus, Aurora, CO, United States; ^2^University of Texas Houston – McGovern Medical School, Houston, TX, United States; ^3^New Health Sciences Inc., Boston, MA, United States; ^4^University of California Berkeley, Berkeley, CA, United States

**Keywords:** hypoxia, metabolomics, mass spectrometry, tracing experiments, flux analysis

## Abstract

State-of-the-art proteomics technologies have recently helped to elucidate the unanticipated complexity of red blood cell metabolism. One recent example is citrate metabolism, which is catalyzed by cytosolic isoforms of Krebs cycle enzymes that are present and active in mature erythrocytes and was determined using quantitative metabolic flux analysis. In previous studies, we reported significant increases in glycolytic fluxes in red blood cells exposed to hypoxia *in vitro* or *in vivo*, an observation relevant to transfusion medicine owing to the potential benefits associated with hypoxic storage of packed red blood cells. Here, using a combination of steady state and quantitative tracing metabolomics experiments with ^13^C_1,2,3_-glucose, ^13^C_6_-citrate, ^13^C_5_^15^N_2_-glutamine, and ^13^C_1_-aspartate *via* ultra-high performance liquid chromatography coupled on line with mass spectrometry, we observed that hypoxia *in vivo* and *in vitro* promotes consumption of citrate and other carboxylates. These metabolic reactions are theoretically explained by the activity of cytosolic malate dehydrogenase 1 and isocitrate dehydrogenase 1 (abundantly represented in the red blood cell proteome), though moonlighting functions of additional enzymes cannot be ruled out. These observations enhance understanding of red blood cell metabolic responses to hypoxia, which could be relevant to understand systemic physiological and pathological responses to high altitude, ischemia, hemorrhage, sepsis, pulmonary hypertension, or hemoglobinopathies. Results from this study will also inform the design and testing of novel additive solutions that optimize red blood cell storage under oxygen-controlled conditions.

## Introduction

Approximately 31,000 packed red blood cell (RBC) units are transfused every day in the US alone (1), thus illustrating the importance of RBC transfusion as a life-saving procedure for millions of people around the world. One hundred years of advancements in the field of transfusion medicine [as reviewed here (2, 3)] have tackled many of the issues associated with making ~110 million units/year available for transfusion all over the world. Though logistically inevitable, refrigerated storage of packed RBCs in the blood bank results in the progressive accumulation of a series of biochemical and morphological alterations, collectively termed the “storage lesion” (4–6). Hallmarks of the storage lesion include the early onset of an impaired energy and redox metabolism (7), which in turn affects redox homeostasis of proteins (8–10), lipids (11–13), and various small molecule metabolites (13–15). Reassuringly, evidence from randomized clinical trials [RCTs—extensively reviewed by Belpulsi and colleagues (16)] suggests that the general standard of care would not be improved by exclusively issuing fresh RBCs, at least for the clinical indications addressed by, and within the statistical power of, the completed RCTs. One tentative explanation reconciling the lack of correlation between the well-established storage lesion and the RCT results could involve the underappreciated role that donor and recipient biology plays in mediating transfusion safety and efficacy (17). In the last 7 years, such large-scale studies as the Recipient Epidemiology and Donor Evaluation Study-III have addressed the issue of biological variability and found that biological variability across donors (i.e., donor ethnicity, gender, and age) affects RBC storability and stress hemolysis (18). Such observations have been supported by smaller scale laboratory studies in humans (19, 20) that demonstrated heritability of the metabolic storage lesion (21–23), as well as studies performed in mice (24, 25) showing that post-transfusion recoveries are greatly variable across donors (26). Of note, Yoshida and colleagues have recently provided preliminary evidence suggesting that hemoglobin oxygen saturation (SO_2_) at 8 h from donation and routine processing varies significantly across donors (27), potentially contributing to the donor-dependent development of the storage lesion. This is relevant in light of accumulating evidence suggesting that SO_2_ significantly impacts RBC metabolism, as is the case in exposure to high-altitude hypoxia or hemorrhagic hypoxia (28, 29), as well as hypoxic storage in the blood bank (30–32). Hypoxic storage boosts energy metabolism and limits oxidative challenge to stored RBC proteins (10, 33), a phenomenon in part explained by the intracellular alkalinization accompanying the simultaneous removal of oxygen and carbon dioxide from the unit (34), as well as by the oxygen-dependent metabolic modulation of glycolytic enzyme activity (10, 35–37). Some of the benefits of anaerobic storage can indeed be phenocopied by alkaline additives (38, 39), which have been shown to boost glycolysis, Rapoport-Luebering shunt and pentose phosphate pathway activation (40) through a positive pH-dependent regulation of phosphofructokinase, bisphosphoglycerate mutase, and glucose 6-phosphate dehydrogenase (2). Because beneficial effects of metabolic interventions to attenuate the storage lesion have been demonstrated by washing and/or rejuvenating end-of-storage erythrocytes (41), boosting RBC metabolism through a combination of SO_2_ control and novel additive solutions may represent a viable strategy to tackle the storability issue and further improve RBC storage quality in the future. Understanding how erythrocyte metabolism is affected by normoxia and hypoxia *in vivo* and *ex vivo* under refrigerated conditions is key to the development of novel additive solutions tailored to packed RBCs stored under oxygen-controlled conditions. In this view, it is worth considering how recent advancements in proteomics have expanded our understanding of the RBC proteome complexity, which was thought to include ~750 proteins just a decade ago (42) and is now known to enlist ~2,800 (43) and counting (44). While identification of trace levels of an enzyme in RBCs does not necessarily imply that the enzyme is functionally active, it has been recently demonstrated through flux experiments using stable isotope tracers that cytosolic isoforms of Krebs cycle enzymes are present and active in mitochondria-devoid human erythrocytes (44), an observation that is relevant for the RBC metabolism of citrate when stored in the most common additives in Europe [SAGM (45, 46)] and in the US [e.g., AS-3 (13)]. In these studies, it was shown that citrate metabolism can contribute to a varying percentage of lactate generation during storage progression (13, 45, 46). Since hypoxia promotes glycolysis and lactate generation in a SO_2_-dependent fashion (10), we hypothesized that carboxylic acid metabolism (including citrate metabolism) in mature RBCs may be affected by hypoxia *in vivo* and *ex vivo* during short term (24 h) and prolonged refrigerated storage (up to 42 days) under SO_2_-controlled conditions. To test this hypothesis, we re-analyzed RBCs from individuals exposed to high-altitude hypoxia to specifically look for carboxylates, as an expansion of the AltitudeOmics study (28). Moreover, we performed integrated metabolic tracing experiments in the presence of different stable isotope-labeled substrates (citrate, glucose, aspartate, and glutamine) in order to determine how hypoxia affected RBC metabolism of these substrates under normoxic and hypoxic conditions.

## Materials and Methods

Blood samples were collected from healthy donor volunteers upon receiving written informed consent and in conformity with the Declarations of Helsinki under protocol approved by the University of Texas Houston and University of Colorado Denver institutional review boards (no. AWC-14-0127 and 11-1581, respectively). Commercial reagents were purchased from Sigma-Aldrich (Saint Louis, MO, USA) unless otherwise noted.

### Human RBCs, Stored under Normoxic or Hypoxic Conditions

Blood was collected from healthy donors at the Bonfils Blood Center (Denver, CO, USA) according to the Declaration of Helsinki. Filter leukocyte-reduced (>99.95% WBC depleted—Pall Medical, Braintree, MA, USA) packed RBCs were stored in CP2D-AS-3 (*n* = 4; Haemonetics Corp., Braintree, MA, USA). Units were sterilely sampled (0.1 mL per time point) on a weekly basis until storage day 42, and cells and supernatants were separated by centrifugation at 2,000 × *g* for 10 min at 4°C.

### High-Altitude Studies

Whole blood was collected from 12 male and 9 female healthy human volunteers at sea level or after 3 h (ALT1 am), >8 h (ALT1 pm), or 7 days (ALT7) of exposure to high-altitude hypoxia (5,260 m) in Mt. Chacaltaya, Bolivia, within the framework of the AltitudeOmics study (28). RBCs were separated from whole blood through gentle centrifugation (~99% WBC depleted), as described (28).

### Labeling Experiments

#### ^13^C_1,2,3_-Glucose and RBC Storage under Controlled Oxygen Saturation Conditions

Filter leukocyte-reduced (>99.95% WBC depleted—Pall Medical, Braintree, MA, USA) packed red blood cells (*n* = 4) were collected, processed, and stored in CP2D-AS-3, as described above, supplemented with additional 11 mM ^13^C_1,2,3_-glucose (no. CLM-4673-PK—Cambridge Isotope Laboratories Inc.—Tewksbury, MA, USA) prior to storage at six different oxygen saturation conditions, monitored throughout storage duration—including controls (untreated—averaging SO_2_ = 47 ± 20), hyperoxic (SO_2_ > 95%), and hypoxic (SO_2_ = 20%, 10%, 5%, or <3%), as previously described (10, 27).

#### Tracing Experiments from Heavy Citrate, Glutamine Aspartate, and Glucose in Hypoxia and Normoxia for 24 h

Filter leukocyte-reduced (>99.95% WBC depleted—Pall Medical, Braintree, MA, USA) RBCs (*n* = 3) were stored for up to 24 h under normoxia (PO_2_ = 21%) or hypoxia (PO_2_ = 8%) in CP2D-AS-3 prepared in house (four independent experiments) in the presence of U-^13^C-glucose (55 mM—Sigma-Aldrich Catalog no. 389374), ^13^C_6_-citric acid (Sigma-Aldrich Catalog no. 606081*—*2.2 mM), ^13^C_1_-aspartate (Sigma-Aldrich Catalog no. 489972—1 mM), or ^13^C_5_^15^N_2_-glutamine (Sigma-Aldrich Catalog no. 607983—4 mM).

### Sample Processing

Packed RBCs and supernatants were extracted in ice cold extraction solution (Optima LC-MS grade methanol:acetonitrile:water 5:3:2 v/v) at 1:10 or 1:25 dilutions, prior to vortexing for 30 min at 4°C. Insoluble proteins were pelleted by centrifugation at 4°C for 10 min at 10,000 × *g* and supernatants were collected and stored at −80°C until subsequent analysis.

### UHPLC-MS Metabolomics Analysis

Sample extracts were analyzed by UHPLC-MS, as previously reported (47). Briefly, analyses were performed on a Vanquish UHPLC system (Thermo Fisher Scientific, San Jose, CA, USA) coupled online to a Q Exactive mass spectrometer (Thermo Fisher Scientific, Bremen, Germany). Samples were resolved over a Kinetex C18 column, 2.1 mm × 150 mm, 1.7 µm particle size (Phenomenex, Torrance, CA, USA) at 25°C using an isocratic runs with 5% B for 3 min at 250 μl/min or a 9 min method from 5 to 95% B flowed at 450 µl/min and 30°C, where mobile phase A consisted of water + 0.1% formic acid (for positive mode) or 5 mM ammonium acetate (for negative mode) and mobile phase B consisted of acetonitrile water + 0.1% formic acid (for positive mode) or 5 mM ammonium acetate (for negative mode). The mass spectrometer was operated independently in positive or negative ion mode scanning in Full MS mode (2 μscans) at 70,000 resolution from 60 to 900 *m/z*, with electrospray ionization operating at 4 kV spray voltage, 15 shealth gas, 5 auxiliary gas. Calibration was performed prior to analysis using the Pierce™ Positive and Negative Ion Calibration Solutions (Thermo Fisher Scientific). Acquired data was converted from .raw to .mzXML file format using Mass Matrix (Cleveland, OH, USA). Metabolite assignments, isotopologue distributions and correction for expected natural abundance of 13C and 15N isotopes were performed using MAVEN (Princeton, NJ, USA) (48).

Graphs were plotted and statistical analyses (either *T*-test or repeated measures ANOVA) performed with GraphPad Prism 5.0 (GraphPad Software, Inc., La Jolla, CA, USA). Significance was assessed through repeated measure ANOVA (time course), two way-ANOVA (SO_2_ conditions), and *T*-test (% isotopologue enrichment)—threshold being *p* < 0.05.

## Results

### High-Altitude Hypoxia Affects Steady-State Levels of Carboxylates in Human RBCs

**Figure 1:**
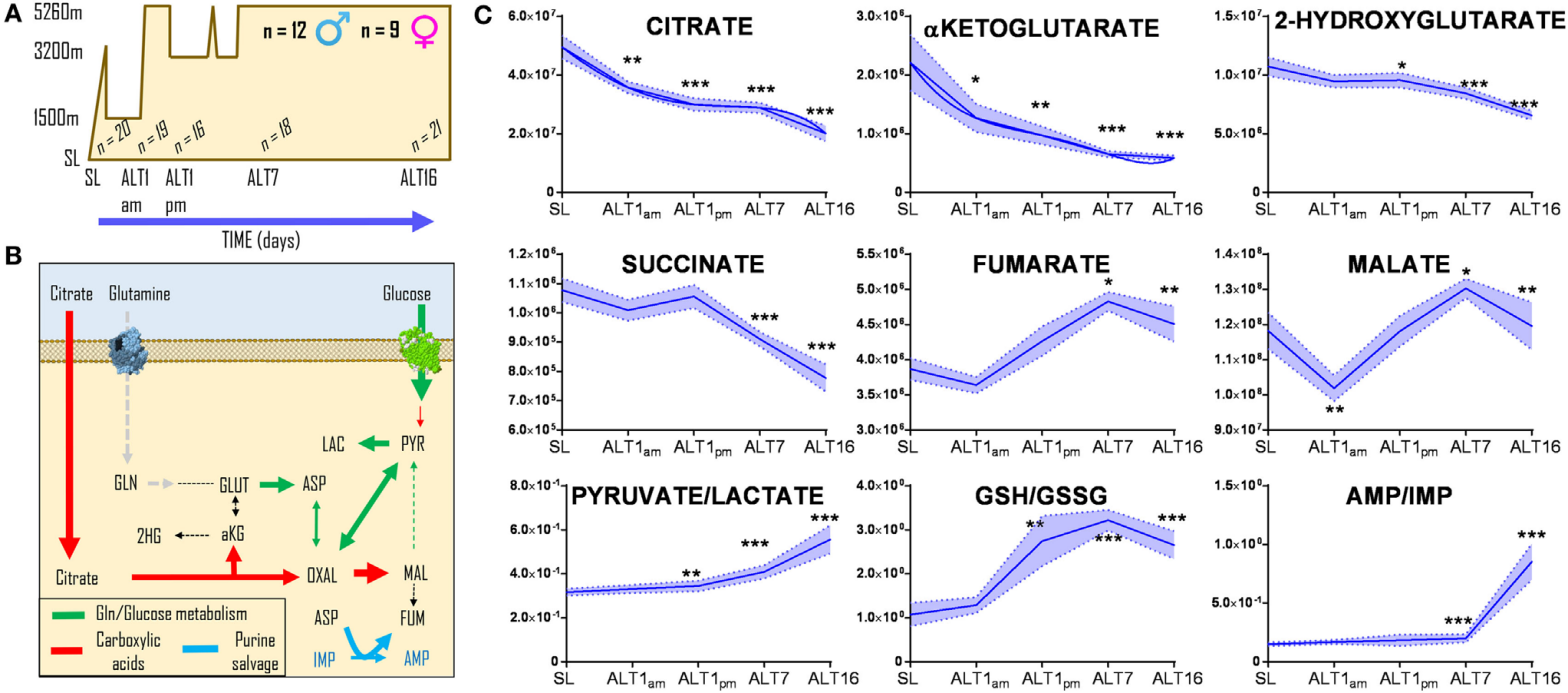
Acclimatization to high-altitude hypoxia decreases steady-state levels of carboxylic acids in human red blood cells. Twenty-one healthy volunteers (12 male and 9 female) were flown from sea level (Oregon) to Bolivia (>5,260 m) for up to 16 days **(A)**, within the framework of the AltitudeOmics study (28, 29). While all of them successfully acclimatized to high-altitude hypoxia (28, 29), red blood cell (RBC) levels **(B)** of citrate, alpha-ketoglutarate, hydroxyglutarate, and succinate decrease from sea level to high altitude, proportionally to the duration of stay at over 5,000 m. Transient decrease and progressive increases in fumarate and malate were observed, paralleled by increases in the pyruvate/lactate ratios and reduced/oxidized glutathione (GSH/GSSG) ratios, suggestive of a progressively more reducing environment in the cytosol of RBCs from individuals acclimatizing to high-altitude hypoxia. *p < 0.05; **p < 0.01; ***p < 0.001 (repeated measures ANOVA). All data points on *x* axis were tested [*n* for each data point is reported in panel **(C)**].

Red blood cells were collected from 21 healthy volunteers (12 male and 9 female) at sea level (SL—Oregon) or within <3 h (ALT1 noon), 8–12 h (ALT1 pm), 7, or 16 days (ALT7 and ALT16, respectively) of exposure to high-altitude hypoxia in Bolivia (Mt. Chacaltaya, >5,260 m) (Figure 1A), within the framework of the AltitudeOmics study (28, 29). Even though previous metabolomics analyses of these RBCs did not cover carboxylic acids (28), new analyses were performed in light of the recent appreciation of carboxylic acid metabolism in mitochondria-deficient mature erythrocytes (13, 45, 46). Exposure to high-altitude hypoxia resulted in a progressive decrease in the RBC levels of carboxylic acids citrate, alpha-ketoglutarate, and 2-hydroxyglutarate from baseline levels at SL, and proportionally to the duration of stay at high altitude (Figure 1B). Transient decreases within hours after exposure to high altitude and progressive increases after 8–12 h during altitude acclimatization were observed for RBC fumarate and malate (Figure 1B). In parallel, elevated ratios of pyruvate/lactate [a proxy for NADH/NAD + ratios according to the mass action law (49)] and reduced/oxidized glutathione (GSH/GSSG) (Figure 1B) were observed, representing markers of a progressively increased reducing environment in the cytosol of RBCs from individuals acclimatizing to high-altitude hypoxia.

### *Ex Vivo* Preservation of Packed RBCs under Controlled SO_2_ Conditions Promotes Citrate Consumption and Accumulation of Fumarate, Malate, and Alpha-Ketoglutarate

**Figure 2:**
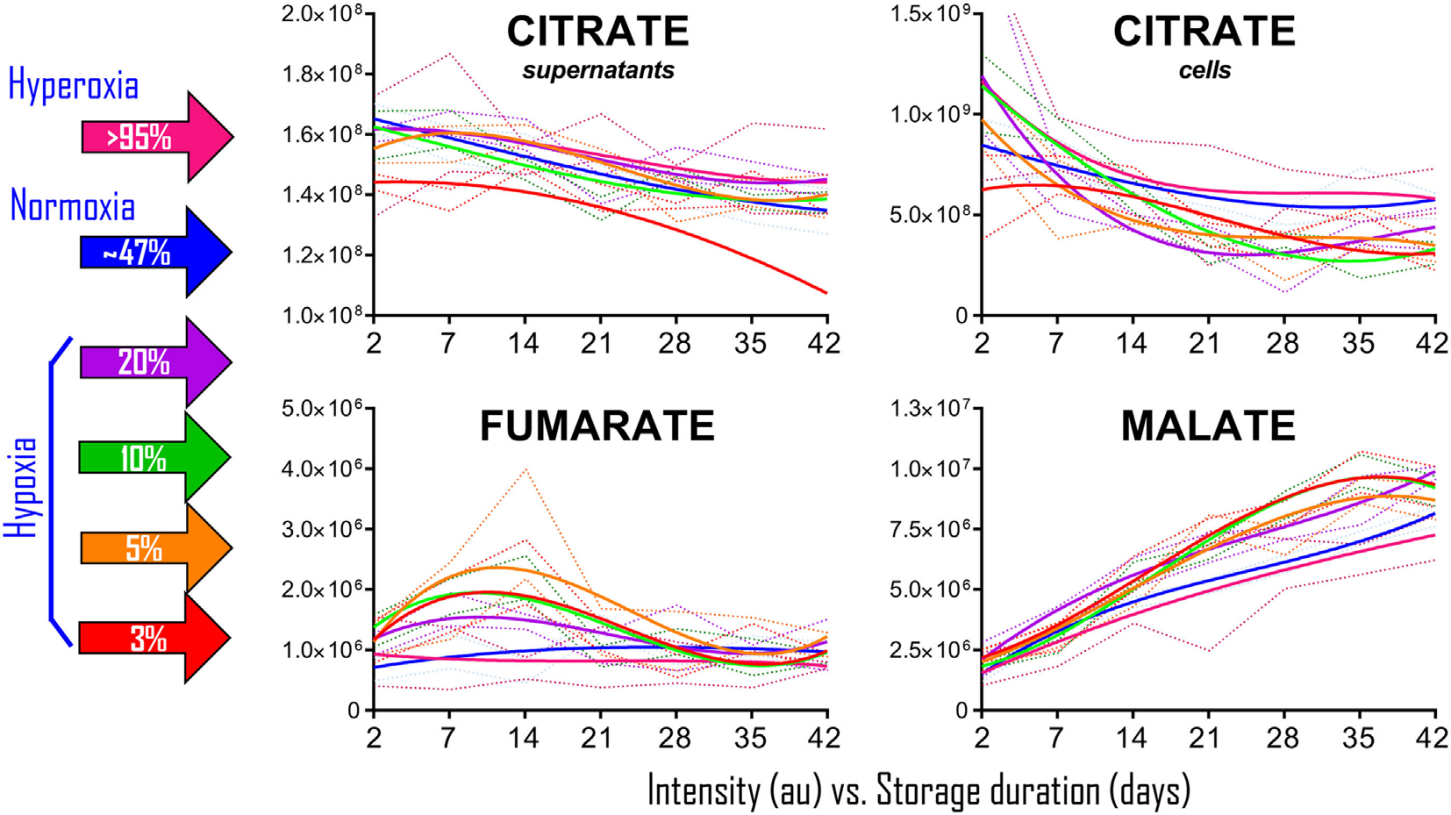
Packed red blood cell (RBC) storage under controlled hemoglobin oxygen saturation conditions recapitulates high-altitude hypoxia-induced decreases in citrate and accumulation of fumarate/malate. RBCs were stored under normoxic conditions (untreated–SO_2_ = 47 ± 21, mean ± SD—solid blue line), hyperoxia (SO_2_ > 95%—solid purple line), or four hypoxic conditions (SO_2_ = 20, 10, 5, or <3%—solid purple, green, orange, and red lines, respectively). Supernatant citrate was significantly lower than controls (*p* < 0.05) in SO_2_ < 3% hypoxic RBCs at all tested time points. Fumarate was significantly higher than controls (*p* < 0.05) at storage day 7 and 14, while malate at day 14 onward in all hypoxic RBCs when compared to controls and hyperoxic counterparts. Dotted lines indicate ranges (same color-code—lighter tone). All data points on *x* axis were tested (*n* = 4).

**Figure 3:**
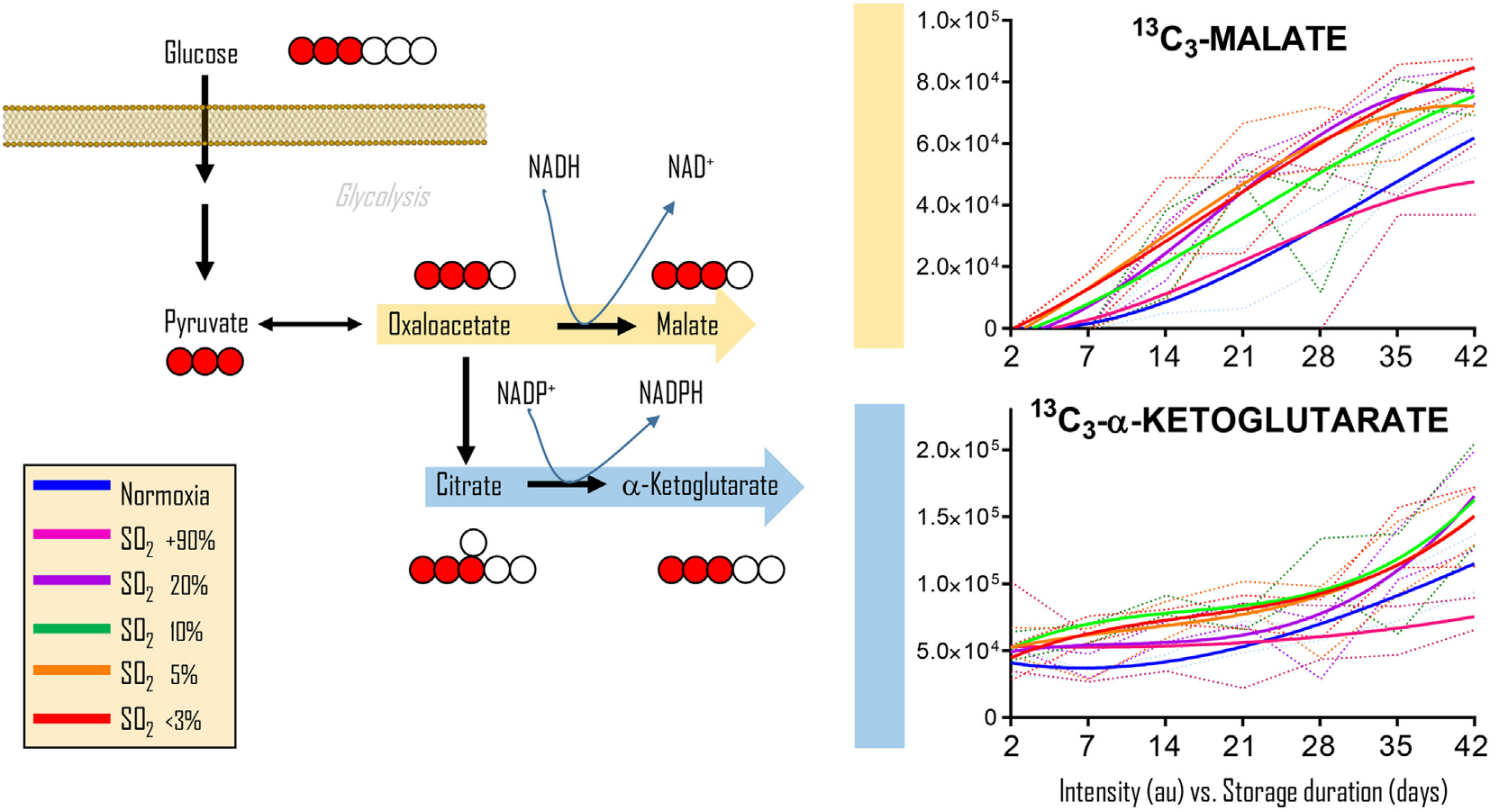
Glucose tracing experiments indicate hypoxia-induced increases in carboxylic acids deriving from glucose. Cytosolic isoforms of Krebs cycle enzymes are present in mature red blood cells (RBCs) and can theoretically catalyze the reactions graphed here, reactions that could contribute to RBC reducing equivalent homeostasis. Heavy fumarate and malate accumulation in hypoxic RBCs was significantly higher (*p* < 0.05) than controls at all tested storage days after day 7. Hyperoxic RBCs had significantly (*p* < 0.05) lower heavy fumarate than control RBCs only at storage day 42. All data points on *x* axis were tested (*n* = 4).

To determine whether the observations in RBCs from individuals exposed to high-altitude hypoxia would be translatable to RBCs stored under oxygen-controlled conditions, we stored RBCs under normoxia (untreated—SO_2_ = 47% ± 21, mean ± SD), hyperoxia (SO_2_ > 95%), or four hypoxic conditions (SO_2_ = 20, 10, 5, or <3%—Figure 2). Citrate consumption proportional to the degree of hypoxia was observed in supernatants and, most notably, in cells during storage in AS-3, therefore suggesting increased consumption of citrate in hypoxic RBCs (Figure 2). In parallel, hypoxic RBCs generated more fumarate for the first 3 weeks of storage, and malate through the whole storage period (Figure 2). Recent proteomics (43, 44), metabolomics (13, 45), and computational evidence (46) has suggested that carboxylate metabolism in mature RBCs can be regulated by enzymatic reactions that are downstream to glucose-derived pyruvate by cytosolic isoforms of Krebs cycle enzymes such as acteyl-coA ligase, phosphoenolpyruvate carboxylase—PEPCK [or PEPCK-like activity of hemoglobin (50)], fumarate hydratase, isocitrate dehydrogenase 1, and malate dehydrogenase 1. To determine whether such reactions were affected by the degree of hypoxia, we incubated RBCs with ^13^C_1,2,3_-glucose under varying SO_2_ conditions (from <3% to >95%) and monitored ^13^C distribution in downstream metabolites according to the reactions summarized in Figure 3. While generation of ^13^C-fumarate from ^13^C_1,2,3_-glucose was not observed, accumulation of ^13^C_3_-malate and ^13^C_3_-alpha-ketoglutarate isotopologues was observed during storage and followed a trend that was inversely proportional to SO_2_ (i.e., higher generation of these compounds from heavy glucose was observed with hypoxia—Figure 3).

### Determination of Isotopologue Distributions upon RBC Exposure to Hypoxia *Ex Vivo* in Presence of Stable Isotope-Labeled Citrate, Glutamine, and Aspartate

**Figure 4:**
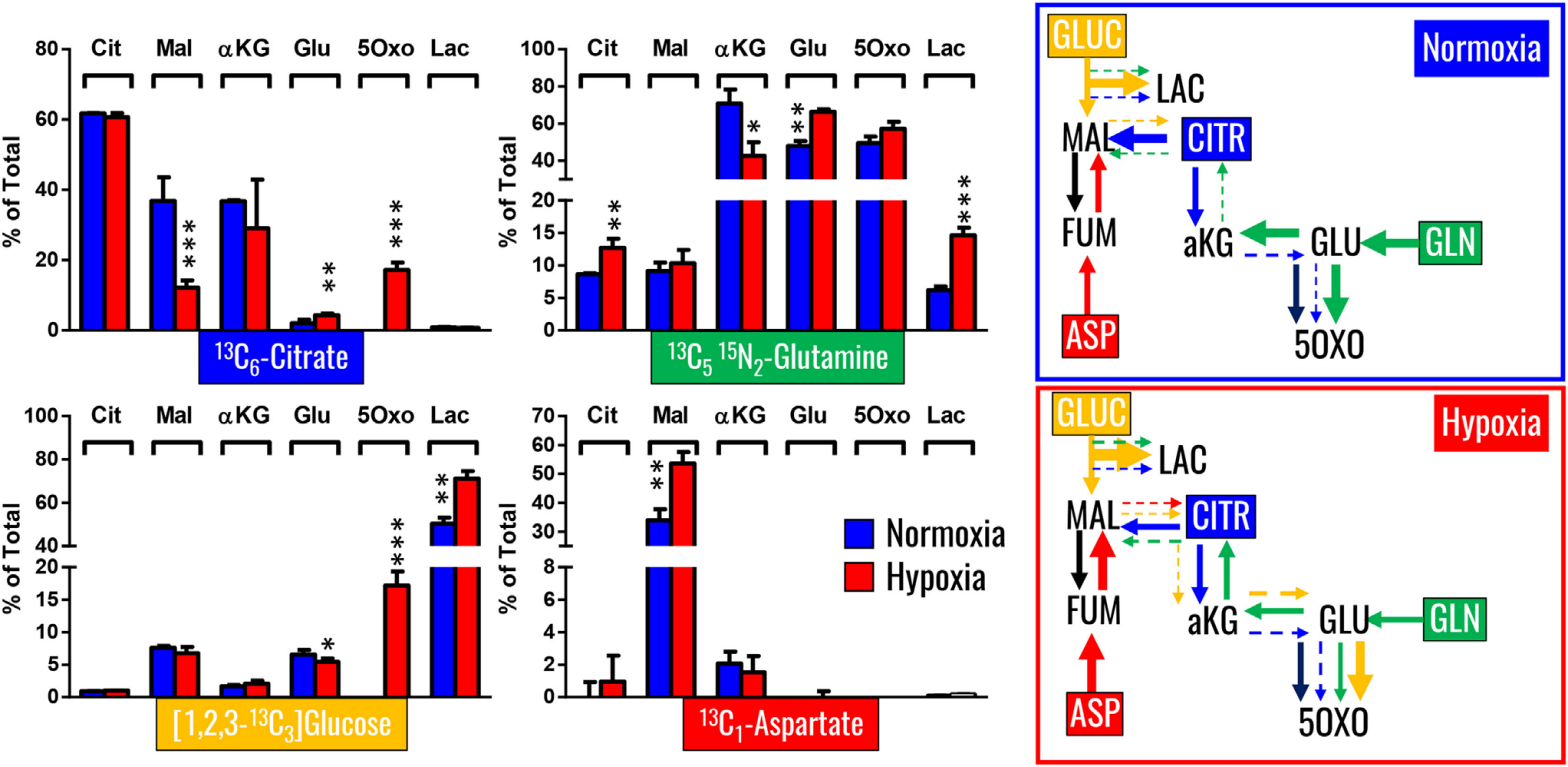
Isotopologue distribution of heavy carbon atoms from heavy citrate, glutamine, glucose, and aspartate indicate a complex rewiring of red blood cell carboxylic acid metabolism in response to hypoxia, as summarized in the panels to the right. Bars indicate median (± SD)% accumulation of heavy isotopologues vs the total levels of the compound, as measured in three independent experiments per each condition (normoxia vs 24 h hypoxia—blue and red bars, respectively). Arrows in the panels to the right indicate metabolic rewiring in normoxia and hypoxia and color-code are consistent with the colors used to identify stable isotope tracers indicated in the four panels to the left. *p < 0.05; **p < 0.01; ***p < 0.001 (*T*-test to normoxic control).

**Figure 5:**
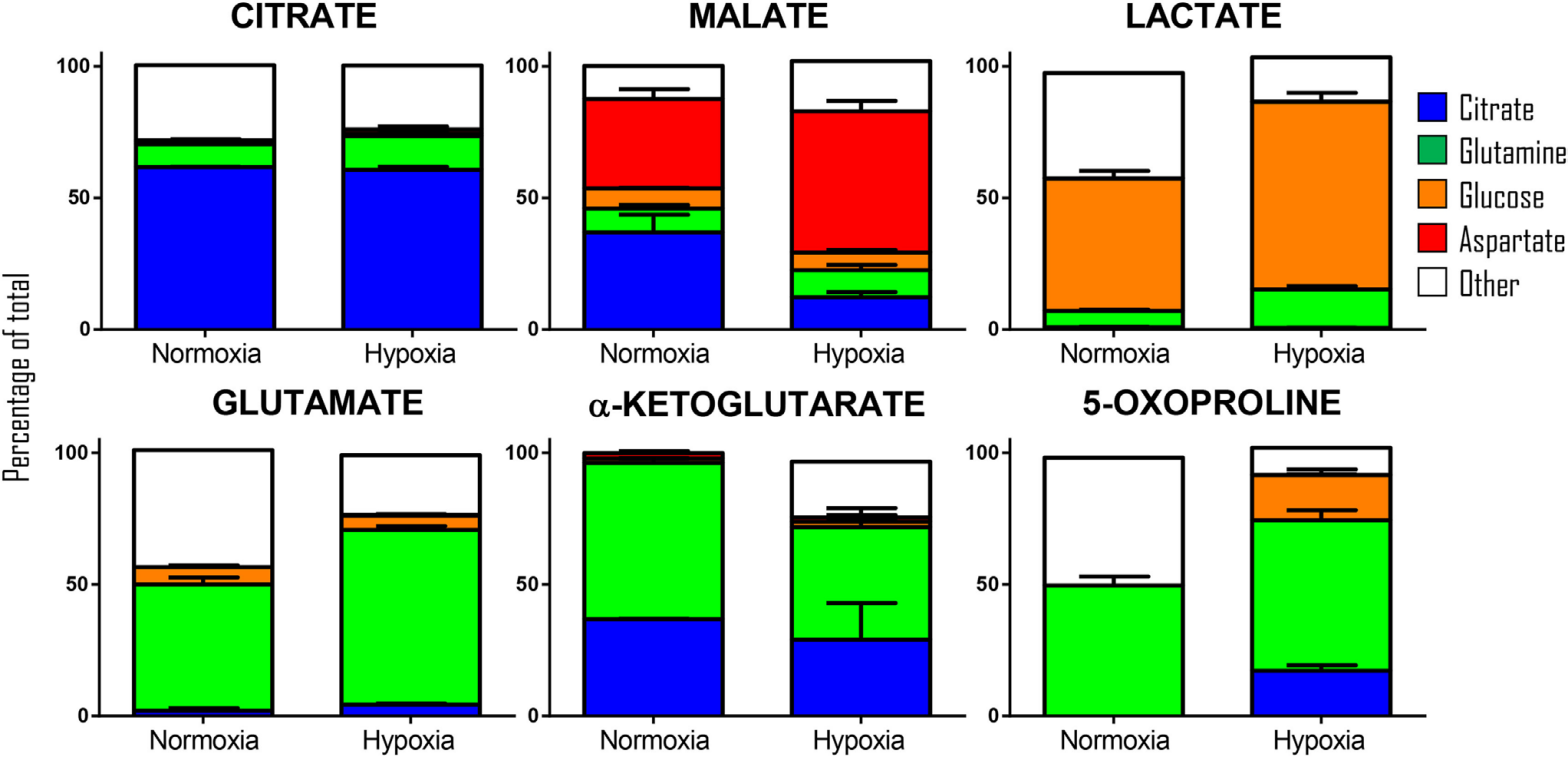
Relative contribution of metabolic substrates (citrate, glutamine, glucoose, aspartate, other) to the generation of citrate, malate, lactate, glutamate, alpha-ketoglutarate, and 5-oxoproline under normoxic or hypoxic conditions (24 h). Mean ± SD are shown from three independent experiments per condition. Other here indicates either endogenous levels of the metabolite or derivation from other sources than the stable isotope tracers used here. Significant increases in glucose-derived lactate and glutamine-derived glutamate, but not ketoglutarate were observed under hypoxic conditions. Citrate and glucose-derived 5-oxoproline increased significantly (*p* < 0.05) under hypoxic conditions.

Our previous results showed encouraging evidence suggesting that the generation of malate and alpha-ketoglutarate from glucose could indeed occur in mature erythrocytes proportional to hypoxia. However, the amount of isotope-contribution was not sufficient to explain the observed increases in steady-state levels of these compounds during hypoxic refrigerated storage (<10% of which were derived from glucose oxidation in both cases of malate and alpha-ketoglutarate). Therefore, we hypothesized that hypoxia-induced catabolism of substrates other than glucose could more completely explain the observed increase in malate and altered metabolism of RBC carboxylic acids. To test this hypothesis, we incubated RBCs for 24 h under normoxic and hypoxic conditions using an in-house generated AS-3 supplemented with U-^13^C-glucose or ^13^C_6_-citratic acid (thereby replacing the unlabeled components in the formulation), ^13^C_1_-aspartate, or ^13^C_5_^15^N_2_-glutamine in four independent experiments (*n* = 3 for each). Heavy isotopologues derived from the catabolism of these substrates were quantified as a percentage of the total levels of the compound of interest, and included carboxylic acids (citrate, malate, and alpha-ketoglutarate), amino acids derived from transmination/oxidation of alpha-ketoglutarate (glutamate, 5-oxoproline), and lactate (Figure 4). In Figures 4 and 5, we provide a bar graph representation of the percent contribution to the generation of the aforementioned compounds from each of the heavy tracers in normoxia and hypoxia. Of note, >60% of RBC citrate was labeled independently from hypoxia, suggesting that the majority of this metabolite is uptaken from the media (Figures 4 and 5). Notably, citrate catabolism to malate was significant under normoxic conditions (~40% of the total) and reduced by hypoxia (<15%), which in turn promoted oxidative citrate metabolism to glutamate and 5-oxoproline (Figure 4). Minimal contribution of citrate catabolism to lactate generation (Figures 4 and 5) was observed under either normoxic or hypoxic conditions for 24 h (<2.5%), suggesting that previous observations in AS-3 (13) may be explained by a metabolic switch only occurring later on during storage. Glutaminolysis mostly fueled the generation of alpha-ketoglutarate and its transamination byproducts glutamate and 5-oxoproline, a phenomenon that was exacerbated by exposure to hypoxia for 24 h (Figure 4). Metabolism of heavy glutamine contributed in part (<10%) to lactate generation under normoxia, and increased under hypoxia (up to 15%) where the contribution of glutamine to citrate reservoirs increased to ~13% of the total (Figures 4 and 5). Glucose catabolism mostly fueled lactate generation (55 to >70% of total lactate after 24 h in normoxia and hypoxia, respectively) and ~18% generation of 5-oxoproline under hypoxic conditions (Figures 4 and 5). Limited glucose incorporation into malate is consistent with tracing experiments with glucose during storage (Figure 3), though hypoxia-triggered increases in glucose metabolism to malate only became apparent after 1 week of storage rather than 24 h (Figures 3 and 4). This is important because we have previously shown that hypoxic RBCs may use glucose-derived carbons to synthesize amino acid moieties necessary for the synthesis of the tripeptide glutathione during hypoxic storage (27). Finally, aspartate catabolism was identified to influence malate generation (<40% under normoxia and up to 60% under hypoxia—Figure 4), making it the main source of hypoxic malate in human RBCs in this study (Figure 5).

## Discussion

Red blood cells are by far the most abundant host cell in the human body, accounting for nearly 80% of the 30 trillion host cells that make up the body of a 175 cm tall 70 kg man (44). Although loaded with hemoglobin (98% of the cytosolic proteome) and devoid of nuclei and organelles, RBCs are far more complex than previously believed (until the last decade or so). Appreciation through proteomics of the presence of cytosolic isoforms of Krebs cycle enzymes in mature erythrocytes has prompted the field to reconsider whether these enzymes are actually active and, if so, whether they actually influence RBC metabolism during routine storage in the blood bank. Indeed, tracing experiments in packed RBCs have suggested that citrate can be metabolized into lactate when stored in SAGM (45) and AS-3 (13); the latter being more directly relevant due to its elevated concentration of citrate (>20 mM) that compensates for the removal of the osmolite mannitol from its formulation. In light of these tracing experiments, it has been suggested that reactions catalyzed by cytosolic isoforms of Krebs cycle enzymes may contribute to the homeostasis of RBC reducing equivalents NADH and NADPH through reactions alternative to glycolysis, pentose phosphate pathway, and methemoglobin reductase, thereby expanding well-established understanding of RBC metabolic networks (51). Refinement of such networks is indeed important for the development of new storage additives, as *in silico* elaboration of quantitative metabolic information of metabolic markers of the storage lesion (52) would help in predicting the metabolic state of RBCs exposed to novel additives (53). In this study, we provide additional information to refine such models by determining the metabolic effect of RBC SO_2_ modulation on carboxylate metabolism. Decreased RBC levels of 2-hydroxyglutarate and succinate in response to high-altitude acclimatization are relevant in that these metabolites are well-established markers of tissue hypoxia [e.g., ischemic (54) and hemorrhagic hypoxia (55)]. In nucleated cells, succinate accumulation is interesting given that it promotes the stabilization of hypoxia inducible factor 1α by inhibiting prolyl hydroxylase, therefore promoting acclimatization responses to hypoxia (56). Since all the subjects enrolled in the AltitudeOmics study effectively acclimatized to high-altitude hypoxia (57), it is interesting to note that declining levels of RBC succinate may be a marker of decreased tissue hypoxia as the subjects acclimatized.

For the first time, we provide evidence that exposure to hypoxia *in vivo* or *ex vivo* affects RBC capacity to metabolize (consume or generate) carboxylic acids. Through a combination of metabolic flux experiments using different stable isotope tracers, we confirm that RBCs can uptake carboxylic acids such as citrate and metabolize them into di-carboxylates (e.g., malate) or transamination intermediates (e.g., alpha-ketoglutarate, glutamate, 5-oxoproline) in an SO_2_-dependent fashion. Most notably, we show that malate accumulation during storage and the exacerbation of this phenomenon under hypoxia are potentially explained by varying metabolic mechanisms, in that aspartate catabolism predominantly contributes to malate generation under hypoxia, rather than glucose or citrate catabolism. In this view, it is interesting to speculate that purine catabolism [deamination of purines to hypoxanthine and xanthine, a well-documented phenomenon in stored erythrocytes (7, 14, 15, 25, 52, 58)] may be influenced by hypoxia. Indeed, aspartate consumption *via* purine salvage reactions would explain increased fumarate accumulation, which in turn would become a substrate for fumarate hydratase [present and active in mature RBCs (46)] for the generation of malate. Future studies will investigate this interesting corollary to the observations reported here. Alternatively, aspartate may represent an eligible substrate (amino group donor) for transamination reactions. This hypothesis is consistent with the observed decrease in the level of alpha-ketoglutarate and increased glutamate isotopologues (both M + 5 and M + 5 + 1). Such observation can only be explained by combined glutamine metabolism to alpha-ketoglutarate (carbon backbone + 5), which is turn transaminated back to glutamate *via* glutamate oxaloacetate transaminases, previously identified in mature RBC proteomics datasets (43, 44).

Finally, though merely observational, the present study provides interesting hypothesis-generating evidence to investigate why carboxylic acid metabolism may be affected by hypoxia in an enucleated cell incapable of *de novo* protein synthesis, as is the case with RBCs. It is fascinating to speculate that, in similar fashion to the oxygen-dependent metabolic modulation model (28, 29, 35–37), post-translational modifications such as phosphorylation mediated by adenosine/AMPK-dependent signaling (59)—recently identified to contribute to hypoxic adaptations in eukaryotes as simple as *S. cervisiae* (60)—may influence enzyme sub-cellular compartmentalization, formation of multi-protein complexes, and activity. RBC multi-enzyme protein complexes have been preliminarily described in mature RBCs and reported to be susceptible to the storage lesion (61). Therefore, it remains to be assessed whether some of the observations reported here could be attributed to factors other than hypoxia-driven intracellular alkalinization that affects the activities of many RBC cytosolic enzymes, such as sub-cellular compartmentalization (e.g., membrane vs cytosol) or oligomerization of Krebs cycle enzymes into alternative multi-protein complexes under hypoxic conditions. Last but not least, the results presented here may be also interpreted as a result of as of yet uncharacterized reactions involving alternative to Krebs cycle cytosolic isoforms. A paradigmatic example of this notion is the conversion of late glycolytic trioses to oxaloacetate, an intermediate in malate/citrate generation/consumption in mature erythrocytes and a reaction that could be catalyzed by hemoglobin (50) through moonlighting functions (62). Similar considerations could be made for other carboxylates such as 2-hydroxyglutarate, which could be generated by lactate dehydrogenase under hypoxic conditions (63). Therefore, future studies will be necessary to disentangle and possibly identify new metabolic networks that are modulated by oxygen level in RBCs.

## Ethics Statement

The AltitudeOmics study has been approved by the University of Colorado Institutional Review Board, Protocol no. 11-1581.

## Author Contributions

TN, JR, KH, and ADa performed metabolomics analyses and plotted the results. KS, EW, AW, and YX generated samples for *ex vivo* tracing experiments. RR designed, performed, and provided samples for high-altitude studies. TY and ADu generated technology and samples for *ex vivo* oxygen-controlled preservation of packed RBCs. ADa wrote the first draft of the manuscript, and all the authors critically contributed to its finalization.

## Conflict of Interest Statement

The authors would like to disclose that TY and ADu are part of New Health Sciences Inc. ADa, KH, and TN are part of Omix Technologies Inc. AD is a consultant for NHSi.
